# *Terminalia* spp.: A Potential Material and Its Limitations in Medicine

**DOI:** 10.21315/mjms2023.30.6.17

**Published:** 2023-12-19

**Authors:** Le Pham Tan Quoc

**Affiliations:** Institute of Biotechnology and Food Technology, Industrial University of Ho Chi Minh City, Ho Chi Minh City, Vietnam

Dear Editor,

I have read the review article entitled ‘Bacterial foodborne illness in Malaysia: *Terminalia* spp. as a potential resource for treating infections and countering antibiotic resistance’ with great interest (1). The authors highlight the therapeutic phytochemicals and antibacterial properties possessed by these species. *Terminalia* spp. are widely cultivated in the tropics, particularly in Southeast Asia and are primarily known as ornamental shade plants. Therefore, the application of these plants in medicine is quite limited. Extracts are mainly isolated from leaves, bark, seeds, roots and fruits using various solvents, including chloroform, methanol and ethanol. However, a critical issue arises from the lack of recorded extraction efficiency in almost all studies. Determining the yield would allow for the selection of appropriate raw material sources and the implementation of suitable planning measures. Notably, previous studies have often utilised stem bark as extraction material, despite its rigidity and the complex pre-treatment processes involved compared to other plant parts. As a result, the achieved yields may fall below the desired levels. In addition, the extraction of bark may lead to the death of the tree, which is environmentally unsustainable. In a specific case involving *T. nigrovenulosa*, bark extracts demonstrated higher extraction yields than leaves when various solvents, such as methanol, ethanol, acetone and water, were employed (2). In general, leaves, seeds, or fruits of *Terminalia* spp. are also optimal choices for extraction due to their significant biological effects (3–5).

Indeed, *T. catappa* has been extensively studied due to its widespread distribution in various regions of the world. Extracts obtained from *T. catappa* leaves have shown a high content of bioactive compounds, such as glycosides, saponins, steroids, alkaloids and polyphenols. These extracts have demonstrated inhibitory effects against several pathogenic bacteria, including *Escherichia coli*, *Bacillus cereus*, *Staphylococcus aureus*, *Pseudomonas aeruginosa*, *Proteus mirabilis*, etc. Akharaiyi et al. (6) found that the *T. catappa* leaf extract exhibited effective minimum inhibitory concentration (MIC) values ranging from 100 mg/mL to 350 mg/mL against the tested microorganisms. Studies investigating the antibacterial properties of other *Terminalia* spp. are limited, particularly in relation to the MIC method (1). However, some *Terminalia* spp. have also shown excellent antibacterial properties; for example, the aqueous extract of *T. chebula* fruit exhibited low MIC values of 195 μg/mL and 390 μg/mL against *P. aeruginosa* and *P. mirabilis*, respectively (7). Additionally, the sequential aqueous extract of *T. bellirica* fruit displayed inhibitory effects against *P. aeruginosa* (MIC = 1 mg/mL–2 mg/mL) and *E. coli* (MIC = 4 mg/mL) (5). These findings suggest that *Terminalia* species are a promising material for further exploration of their biological properties.

In contrast, the extraction method has received limited attention in previous studies. Typically, researchers have employed a simple immersed process at room temperature without investigating key extraction parameters, such as temperature, pressure, time and solvent-to-material ratio. It is well known that the chemical composition and biological activity of an extract are strongly influenced by the extraction process. Indeed, many new extraction methods have emerged, such as microwave-assisted extraction, ultrasound-assisted extraction, enzyme-assisted extraction, supercritical fluid extraction, etc. These methods have demonstrated the potential to improve extraction efficiency, save time and reduce costs. It is advisable to incorporate these advanced techniques in future research on *Terminalia* spp. extracts.

Another aspect worth considering is the study of essential oil (EOs) derived from *Terminalia* spp., although the yield of EOs is generally low; for example, the EO yield from the leaves and fruits of *T. arjuna* was reported to be 0.2% (8), while in the case of *T. mantaly*, the EO yields from leaves, stem-bark and twigs were found to be 0.28%, 0.13% and 0.11%, respectively (9). It is noteworthy that the authors of these studies solely employed the distillation method for EO extraction without utilising pretreatment techniques, such as enzymatic or ultrasound treatment. Implementing such pretreatment methods can disrupt plant cells and potentially enhance the efficiency of distillation.

Furthermore, an important concern that requires attention is the potential toxicity of EOs and extracts derived from *Terminalia* spp. to humans. Thus far, previous studies have only indicated toxicity in animal models, specifically rats, carp, goldfish and tiger barbs (10, 11). Therefore, there is an evident requirement for conducting clinical studies on humans to address this concern.

One of the most intriguing components of *Terminalia* spp. is the seed oil. Although nearly 300 *Terminalia* species exist, we have primarily focused on the seed oil extracted from *T. belerica* and *T. catappa*. The seed oil yields from these species are considerable, with *T. belerica* yielding approximately 31% and *T. catappa* yielding approximately 56% (12, 13). These oils are rich in both saturated fatty acids, such as palmitic acid and unsaturated fatty acids, including oleic and linoleic acid (4, 12), which are essential for human health. Recent research on *T. catappa* seed oil has revealed its potent antioxidant activity (IC_50_ = 950 μg/mL) as well as antibacterial properties. In one study, the oil exhibited antibacterial activity against five strains: i) *B. cereus*, ii) *S. aureus*, iii) *E. coli*, iv) *P. aeruginosa* and v) *Vibrio parahaemolyticus* (4). These findings highlight the valuable potential of *T. catappa* seed oil as an alternative ingredient in food and medicine.

However, there remains a need to explore the chemical composition, oil content and biological properties of other *Terminalia* spp. Additionally, understanding how these properties affect the human body requires further investigation. Addressing these questions through subsequent studies is crucial, as it highlights a gap in scientific knowledge and underscores the potential of *Terminalia* spp. for medicinal purposes in the future.

In general, studying the biological activity of *Terminalia* species is a complex task due to the diverse number of species and their intricate chemical composition. This letter points out the limitations and potential directions for future research, motivating scientists to focus on improving knowledge and addressing the shortcomings in this field.
